# Postural and Head Control Given Different Environmental Contexts

**DOI:** 10.3389/fneur.2021.597404

**Published:** 2021-06-03

**Authors:** Anat V. Lubetzky, Jennifer L. Kelly, Bryan D. Hujsak, Jenny Liu, Daphna Harel, Maura Cosetti

**Affiliations:** ^1^Department of Physical Therapy, Steinhardt School of Culture, Education, and Human Development, New York University, New York, NY, United States; ^2^Vestibular Rehabilitation, New York Eye and Ear Infirmary of Mount Sinai, New York, NY, United States; ^3^Department of Applied Statistics, Social Science, and Humanities, Steinhardt School of Culture Education and Human Development, New York University, New York, NY, United States; ^4^Department of Otolaryngology-Head and Neck Surgery, New York Eye and Ear Infirmary of Mount Sinai, New York, NY, United States

**Keywords:** sensory integration for postural control, Head Mounted Display, vestibular disorders, hearing loss, balance

## Abstract

Virtual reality allows for testing of multisensory integration for balance using portable Head Mounted Displays (HMDs). HMDs provide head kinematics data while showing a moving scene when participants are not. Are HMDs useful to investigate postural control? We used an HMD to investigate postural sway and head kinematics changes in response to auditory and visual perturbations and whether this response varies by context. We tested 25 healthy adults, and a small sample of people with diverse monaural hearing (*n* = 7), or unilateral vestibular dysfunction (*n* = 7). Participants stood naturally on a stable force-plate and looked at 2 environments via the Oculus Rift (abstract “stars;” busy “street”) with 3 visual and auditory levels (static, “low,” “high”). We quantified medio-lateral (ML) and anterior-posterior (AP) postural sway path from the center-of-pressure data and ML, AP, pitch, yaw and roll head path from the headset. We found no difference between the different combinations of “low” and “high” visuals and sounds. We then combined all perturbations data into “dynamic” and compared it to the static level. The increase in path between “static” and “dynamic” was significantly larger in the city environment for: Postural sway ML, Head ML, AP, pitch and roll. The majority of the vestibular group moved more than controls, particularly around the head, when the scenes, especially the city, were dynamic. Several patients with monaural hearing performed similar to controls whereas others, particularly older participants, performed worse. In conclusion, responses to sensory perturbations are magnified around the head. Significant differences in performance between environments support the importance of context in sensory integration. Future studies should further investigate the sensitivity of head kinematics to diagnose vestibular disorders and the implications of aging with hearing loss to postural control. Balance assessment and rehabilitation should be conducted in different environmental contexts.

## Introduction

The ability to adapt to changes in the sensory environment is considered critical for balance (1). Healthy individuals are able to maintain their balance with their eyes closed, for example, because they will rely on other senses (e.g., vestibular, somatosensory) for postural control (2, 3). An inability to reweight sensory information may lead to loss of balance with environmental changes, e.g., darkness, rapidly moving vehicles (due to visual dependence), or slippery surfaces (due to somatosensory dependence) (2, 4). Historically, the inputs considered for balance consisted of visual, vestibular, and somatosensory, but recent studies suggest that auditory input may serve as a 4th balance input (5). The presence of stationary white noise has been shown to be associated with reduced postural sway, particularly during challenging balance tasks such as standing on foam or closing the eyes (6). To understand the role of sounds in postural control, it is important to combine different levels of auditory and visual cues to better reflect day-to-day postural responses in healthy individuals.

Context has been shown to have an important impact on balance performance, potentially induced by cognitive and emotional aspects, such as postural threats, fear of imbalance or symptoms related to past experiences within specific environments (7). This top-down modulation can interact with the multisensory integration process to affect the motor plan and cannot be captured without providing an environmental context. To facilitate transfer of balance control, it is imperative that we test and train individuals in conditions as close as possible to those commonly encountered during daily activities (8). The importance of context may be analogous for auditory stimuli. While limited research exists on the relationship between auditory input and postural control, a few studies incorporated natural sounds (e.g., a fountain) and suggested that differences in response to natural sounds relate to the properties of the sounds (greater variety of binaural and monaural cues including static and moving features) (9) and the innate emotional/cognitive responses of the individual (5). The majority of studies, reporting that balance is context-dependent, however, refer to the task (single or dual, static or dynamic) or the surface type (10–13). Current virtual reality technology allows context-based testing of multisensory integration and balance using Head Mounted Displays (HMDs) (14).

A novel HMD-based sensory integration paradigm where visual and auditory cues are manipulated in different contexts could be of particular importance to people with sensory loss. Individuals with vestibular dysfunction appear to develop a substitution strategy whereby the remaining sensory inputs (e.g., vision) are weighted more heavily (15–21). Such a strategy is problematic in hectic environments (22). Indeed, individuals with vestibular dysfunction complain of worsening dizziness and balance loss in complex settings such as busy streets (23–25). Data are accumulating regarding the importance of sounds for balance (6). Likewise, several studies suggested an independent relationship between hearing loss, balance impairments and increased risk for falls (26, 27). At present, the mechanism underlying imbalance in patients with hearing loss who do not present with vestibular symptoms is not clear; potential mechanisms include a common inner ear pathology, abnormal sensory weighting/reweighting, cognitive processing or a combination of these (6). Additional research is necessary to explore these and/or other mechanisms mediating imbalance in these groups.

Postural responses to visual perturbations and the contributions of sounds to balance are typically quantified *via* postural sway (6). HMDs, designed to move the virtual scene according to the participant's head movement, accurately (28) record head position at 60–90 Hz with no additional equipment (29). Some studies, however, found differences in head kinematics, and not in postural sway, between patients with visual sensitivity or vestibular dysfunction and controls in response to visual perturbations (30–33). Indeed, people with vestibular loss demonstrated increased head movement compared with controls in response to head perturbations, potentially associated with excessive work of their neck muscles in order to control the head in space (34, 35). In related work we observed that responses to visual cues were magnified at the head segment also among healthy young adults. Head kinematics may provide an important additional facet of postural control beyond postural sway.

The aims of this study were as follows:

1. Determine how postural sway and head kinematics change in healthy adults in response to auditory perturbations when combined with visual perturbations. We expected more movement in response to the visual perturbations, particularly around the head but also for postural sway. Based on prior studies showing a significant reduction in head movement among healthy adults with broadband white noise via speakers (36) or a reduction in head movement with 2 speakers projecting a 500 Hz wave in people who are congenitally blind (37) we hypothesized that head movement will also increase with the sound perturbations.
2. Determine whether the response to sensory perturbations (*via* postural sway and head kinematics) varies by context. Given that balance is known to be context-dependent, we expected the responses to sensory perturbations to be magnified in a semi-real contextual scene.
3. Explore the feasibility of this novel HMD assessment in individuals with vestibular loss and hearing loss and establish a protocol for future research.

## Methods

### Sample

Healthy controls (*N* = 25) were recruited from the University community. Adults with chronic (>3 months) unilateral vestibular hypofunction (*N* = 7) participating in vestibular rehabilitation for complaints of dizziness or imbalance were recruited from the vestibular rehabilitation clinic at the New York Eye and Ear Infirmary of Mount Sinai (NYEEIMS). Adults with monaural hearing (*N* = 7) for various reasons who had no current vestibular issues or self-reported imbalance were recruited from the Otolaryngology clinic at the NYEEIMS. We defined monaural hearing as an ability to hear on one side only due to a single-sided hearing loss or due to bilateral profound hearing loss corrected with a single cochlear implant (CI). Inclusion criteria for all groups were: 18 or older, normal or corrected to normal vision, normal sensation at the bottom of the feet, and ability to comprehend and sign an informed consent in English. This study was approved by the Institutional Review Board of Mount Sinai and by the New York University Committee on Activities Involving Human Subjects.

### System

Visuals were designed in C# language using standard Unity Engine version 2018.1.8f1 (64-bit) (Unity Tech., San Francisco, CA, USA). The scenes were delivered via the Oculus Rift headset (Facebook Technologies, LLC) controlled by a Dell Alienware laptop 15 R3 (Round Rock, TX, USA) with a single sensor placed on a tripod 1.6 meters in front of the participant. The rift has a resolution of 1,080 ×1,200 pixels per eye and uses accelerometers and gyroscopes to monitor head position with a refresh rate of 90 Hz. It has a field of view of 80° horizontal and 90° vertical. Environmental auditory cues were captured with the Sennheiser Ambeo microphone in first order Ambisonics format. The background sounds merged with a sound design process which involved simulating the detailed environmental sounds that exist within the natural environment to develop a real-world sonic representation. Abstract sounds were generated in Matlab. The audio files were processed in Wwise and integrated into Unity.

### Scenes

#### Stars

**Figure 1:**
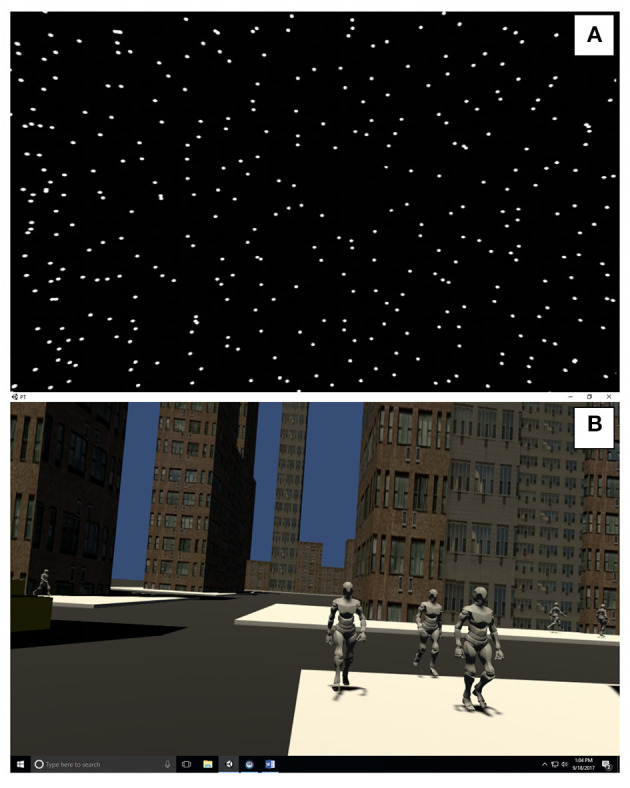
A screenshot of the Stars scene **(A)** and the City scene **(B)**.

The participants observed a 3-wall (front and 2 sides) display of randomly distributed white spheres (diameter 0.02 m) on a black background (See Figure 1A and Supplementary Material) (38). Each wall was 6.16 by 3.2 m with clear central area of occlusion of 0.46 m in diameter to suppress the visibility of aliasing effects in the foveal region (39). Similar to Polastri and Barela (40) the spheres were either static or moving at a constant frequency of 0.2 Hz with either a “low” amplitude (5 mm, AP5) or “high” amplitude (32 mm, AP32). The sounds were developed as rhythmic white noise, scaled to the visual input (Table 1).

**Table 1:** Description of the auditory and visual stimuli in each scene.

**The scene**	**Stars**	**City**
Low visual	0.2 Hz, 0.005 m, in the anterior-posterior direction (AP5)	1–4 avatars are moving from front and back at a speed of 0.51–1.40 m/s.
Low sound	White noise that cycles from 0 to 0.25 dB above played intensity at 0.3 Hz	Ambient sounds include people chatting and city rumbling sounds, mainly caused by traffic.
High visual	0.2 Hz, 0.032 m, in the AP direction (AP32)	4–8 avatars, moving in the same speed and direction as “low.” Ten yellow cars are circling around the street.
High sound	White noise that cycles from 0 to 1 dB above played intensity at 0.3 Hz	Complex sounds include footsteps, car horns honking, a jackhammer, and sirens.
Static, no sound	Display of stars with no movement or sounds	Display of the “high” city with 0 speed (no movement) and no sound.

*Each scene lasted 60 s*.

#### City

The city scene simulates a street with buildings at randomly generated heights, cars and pedestrian avatars (See Figure 1B and Supplementary Material). The difference between “low” and “high” visuals was the amount of avatar pedestrians and the addition of moving cars. Pre-recorded sounds were also scaled “low” and “high” levels of complexity (Table 1).

### Testing Protocol

Participants stood hip-width apart on a stable force-platform wearing the Oculus Rift and were asked to look straight ahead and do whatever felt natural to them to maintain their balance. Participants were guarded by a student physical therapist. Two quad canes were placed on either side of the force-platform for safety and to help with stepping on and off the force-platform. Most participants completed 3 repetitions of each dynamic combination (low, low; low, high; high, low; high, high) and 1 of the “static visuals/no sound” scene per environment. To monitor cybersickness, the Simulator Sickness Questionnaire (SSQ) (41) was administered at baseline, breaks and at the end of the session. The Dizziness Handicap Inventory (DHI), (42) the Activities Specific Balance Confidence Scale (ABC), (24) and a demographic questionnaire were completed during their rest breaks.

### Data Reduction and Outcome Measures

The scenes were 60-s long and the last 55 s were used for analysis (38). Postural sway was recorded at 100 Hz by Qualisys software for a Kistler 5233A force-platform (Winterthur, Switzerland). Head kinematics was recorded at 90 Hz by a custom-made software for the Oculus Rift headset. The criterion validity of the Oculus Rift to quantify head kinematics within postural tasks as compared with a motion capture system has been established (28). We applied a low-pass 4th order Butterworth filter with a conservative cutoff frequency at 10 Hz (43). Directional Path (DP) (44) was calculated as the total path length of the position curve for a selected direction. DP is a measure of postural steadiness and is used as an indication of how much static balance was perturbed with a given sensory manipulation. DP was calculated in 2 directions for force-platform data (AP, ML in mm) and 5 directions of head data [AP, ML in mm, pitch (up and down rotation), yaw (side to side rotation), roll (side flexion) in radians]. DP derived from a force platform is a valid and reliable measure of postural steadiness (45). We previously demonstrated the test-retest reliability of postural sway DP within a similar protocol without the sounds (46).

### Statistical Analysis

#### Aim 1

We generated box plots for each outcome measure (postural sway DP AP and ML and head DP AP, ML, pitch, yaw, roll) per environment across the 5 conditions (Table 1). We conducted a visual inspection of the box plots to determine whether the distributions differed by sensory perturbations among healthy adults.

#### Aim 2

Given that Aim 1 showed complete overlap of the box plots for the 4 dynamic conditions regardless of environment or variable, we combined all dynamic scenes into a single level (dynamic). For each of the 7 variables, we fit a linear mixed effects model (47, 48). Linear mixed-effect models were used to estimate overall differences between environments (Stars, City) and 2 levels of sensory perturbations (static, dynamic) in healthy adults. Based on initial inspection of the residual plots for these models we used a log-transformation of the response variable to limit the impact of heteroscedasticity. These models account for the individual-level variation that is inherently present when repeated measures are obtained from individuals through a random intercept for each individual. No random slopes were used. We present the model coefficients and their 95% confidence interval (CI) for each environment (stars, city) and level (static, dynamic). *P*-values for each fixed effect are calculated through the Satterthwaite approximation for the degrees of freedom for the T-distribution (49). In addition, for ease of clinical interpretation, we provide the estimated marginal means for each of the 4 conditions on the original response scale (mm or radians) along with their confidence intervals. Analyses and figures were created in R Studio version 1.1.423 (50).

#### Aim 3

We used descriptive statistics and inspected violin plots to explore how the patients are distributed around the controls' mean performance. The violin plot depicts the kernel density estimate where the width of each curve corresponds with the frequency of data points in each region. A box plot is overlaid to provide median and interquartile range. Individual data points are represented as black dots.

## Results

For a description of the sample see Table 2.

**Table 2:** Sample demographics.

	**Control**	**Vestibular hypofunction**	**Monaural hearing**
Sex	17 women (68%)	5 women (71%)	2 women (29%)
	8 men (32%)	2 men (29%)	5 men (71%)
Age	Mean 28.40 (SD = 8.48)	Mean 53.7 (SD = 18.0)	Mean 52.57 (SD = 19.50)
DHI	Mean 0 (SD = 0)	Mean 26 (SD = 10.46)	Mean 12 (SD = 18.97)
ABC	Mean 100% (SD = 0)	Mean 74.55% (SD = 18.56)	Mean 90.56% (SD = 16.07)
SSQ baseline	Mean 0.20 (SD = 0.50)	Mean 4.86 (SD = 6.74)	Mean 2 (SD = 2.08)
SSQ final	Mean 0.90 (SD = 1.2)	Mean 7.43 (SD = 6.85)	Mean 2.14 (SD = 3.53)

*DHI, Dizziness Handicap Inventory; ABC, Activities Specific Balance Confidence; SSQ, Simulator Sickness Questionnaire pre- and post-testing*.

### Aim 1

**Figure 2:**
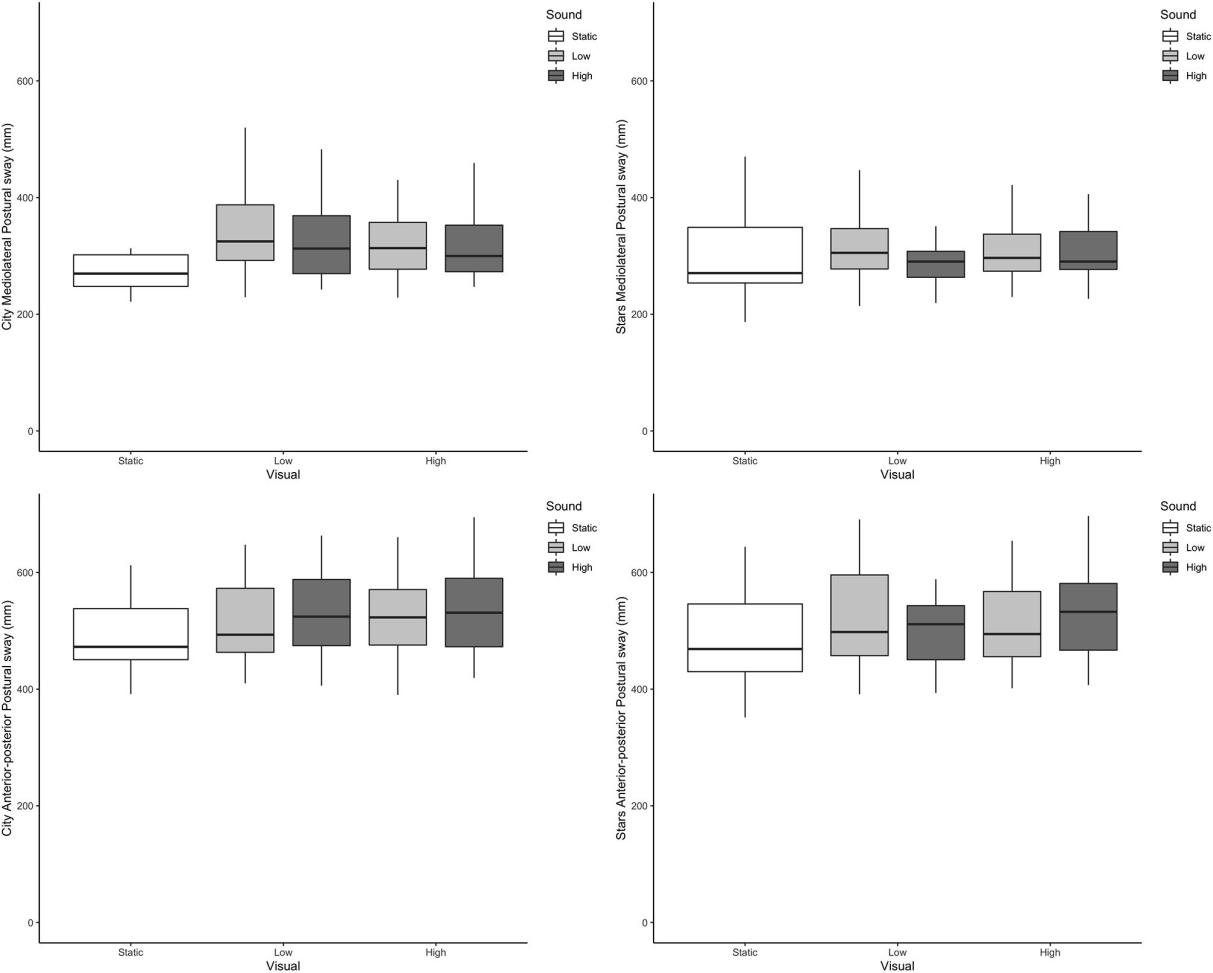
Boxplots of postural sway directional path in mm (Y axis) across the different visual and auditory levels for the city scene (left-hand side) and stars scene (right-hand side) in the medio-lateral direction (top) and anterior-posterior direction (bottom).

**Figure 3:**
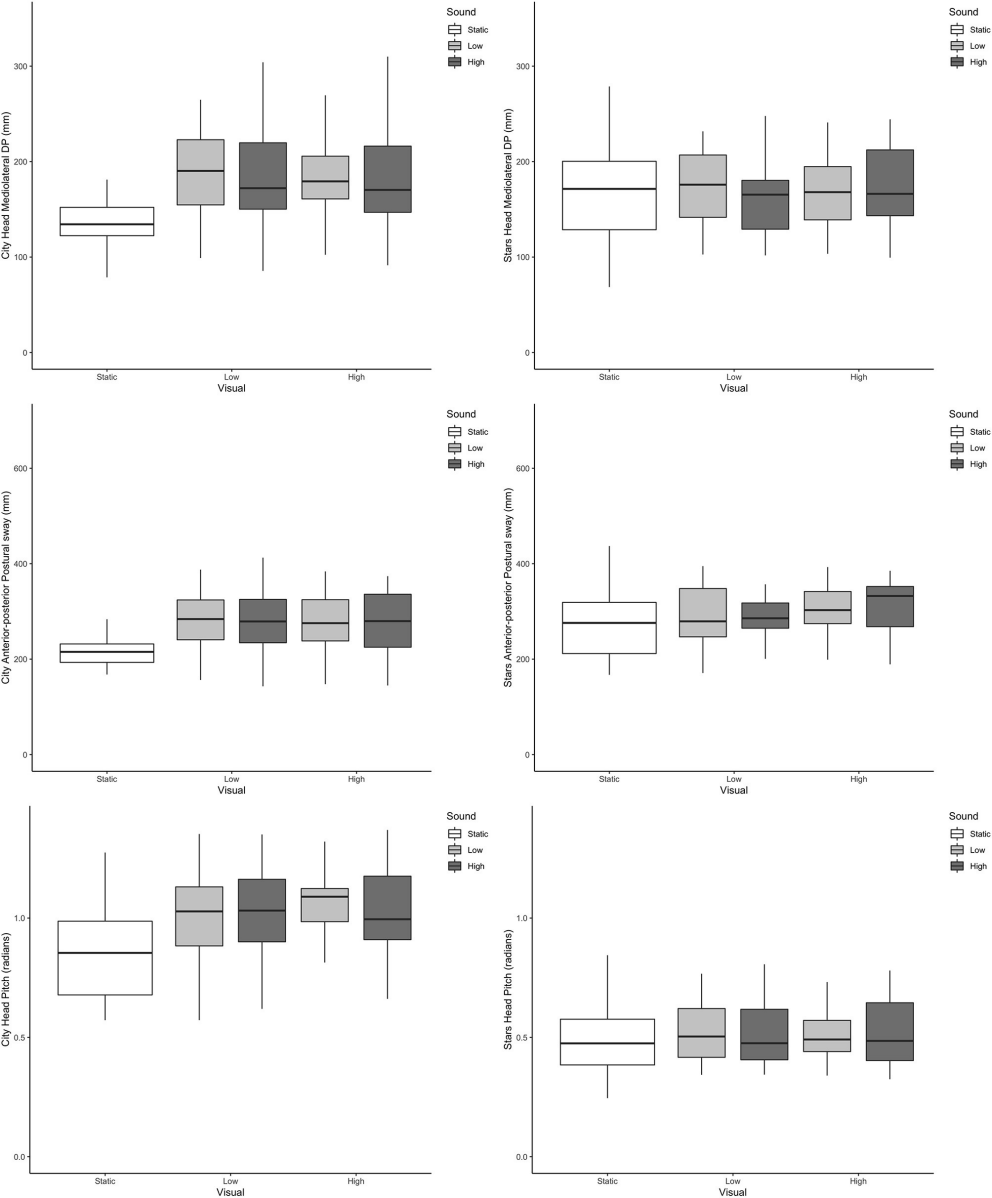
Boxplots of head directional path (Y axis) across the different visual and auditory levels for the city scene (left-hand side) and stars scene (right-hand side) in the medio-lateral direction (top, mm); anterior-posterior direction (middle, mm) and pitch (bottom, radians).

Across all outcome measures and all conditions, box plots of the scenes that included dynamic visual and auditory perturbations showed a complete overlap. Representative examples from postural sway and head data can be seen in Figures 2, 3, respectively.

### Aim 2

Given the lack of difference between “low” and “high” perturbations, we combined all perturbations data into a single “dynamic” category and compared it to the static level. All model coefficients are presented on the log scale whereas estimated marginal means in the response scale are presented in Table 3.

**Table 3:** Directional path estimated marginal means in the response scale with 95% confidence intervals.

	**Stars static**	**Stars dynamic**	**City static**	**City dynamic**
Postural sway ML (mm)	301 (274, 331)	304 (282, 327)	281 (255, 309)	321 (298, 345)
Postural sway AP (mm)	487 (454, 523)	513 (484, 544)	482 (449, 517)	532 (502, 564)
Head ML (mm)	161 (141, 183)	162 (146, 179)	133 (117, 152)	176 (158, 195)
Head AP (mm)	268 (242, 297)	288 (264, 315)	210 (190, 233)	268 (246, 293)
Pitch (radians)	0.47 (0.41, 0.53)	0.50 (0.45, 0.55)	0.87 (0.77, 0.98)	1.11 (1.01, 1.23)
Yaw (radians)	0.37 (0.32, 0.42)	0.40 (0.36, 0.45)	0.76 (0.67, 0.87)	0.91 (0.82, 1.02)
Roll (radians)	0.30 (0.26, 0.34)	0.32 (0.29, 0.36)	0.56 (0.49, 0.63)	0.71 (0.63, 0.79)

#### Postural Sway ML

We observed no significant main effects of sensory perturbations or environment, but a significant sensory perturbation by environment interaction such that the increase in Sway ML was larger between static and dynamic conditions in the city (β = 0.124, 95% CI 0.028, 0.221, *P* = 0.012) compared with the stars.

#### Postural Sway AP

We observed a significant increase in Sway AP between static and dynamic for both scenes (β = 0.051, 95% CI 0.006, 0.096, *P* = 0.025), with no main effect of environment or sensory perturbations by environment interaction.

#### Head ML

There were no significant differences between static and dynamic for Head ML for the stars scene. A significant main effect of environment was observed, such that that Head ML was significantly lower in the city compared with the stars (β = −0.191, 95% CI −0.311, −0.07, *P* = 0.002) and a significant sensory perturbation by environment interaction (β = 0.274, 95% CI 0.148, 0.399, *P* < 0.001) such that there was a significant increase with the dynamic condition in the city.

#### Head AP

We observed a significant increase in Head AP DP between static and dynamic for both scenes (β = 0.073, 95% CI 0.015, 0.13, *P* = 0.013), a significantly lower Head AP DP with city compared with stars (β = −0.244, 95% CI −0.322, −0.166, *P* < 0.001) and a significant sensory perturbations by environment interaction (β = 0.172, 95% CI 0.09, 0.253, *P* < 0.001) such that the increase with the dynamic condition was higher in the city compared with the stars.

#### Head Pitch, Yaw, and Roll

There were no significant main effect of sensory perturbations for pitch, yaw or roll. We observed a significant main effect of environment for pitch (β = 0.628, 95% CI 0.519, 0.737, *P* < 0.001) and roll (β = 0.623, 95% CI 0.521, 0.726, *P* < 0.001) but not for yaw. Significant sensory perturbations by environment interactions were also observed for pitch (β = 0.177, 95% CI 0.063, 0.291, *P* = 0.002) and roll (β = 0.171, 95% CI 0.065, 0.278, *P* = 0.002), but not for yaw.

### Aim 3

**Figure 4:**
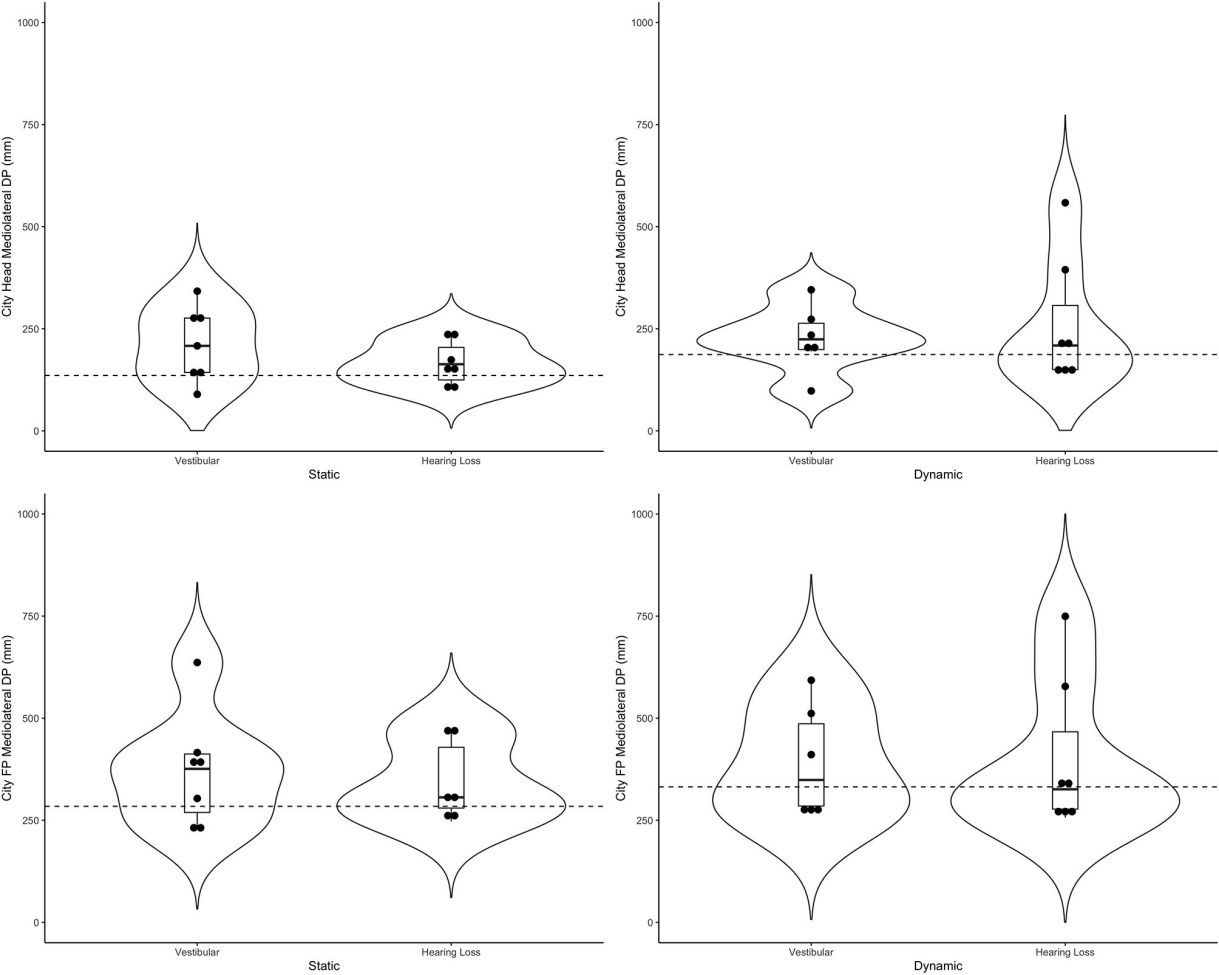
Violin plots representing the distribution of both clinical groups around the mean of the control group (represented by the dashed line) for the city scene in the medio-lateral direction. Top plots show head data and bottom plots represent postural sway data (FP = forceplate). Left-hand side represents the static scenes and right-hand side represents the dynamic scenes.

**Figure 5:**
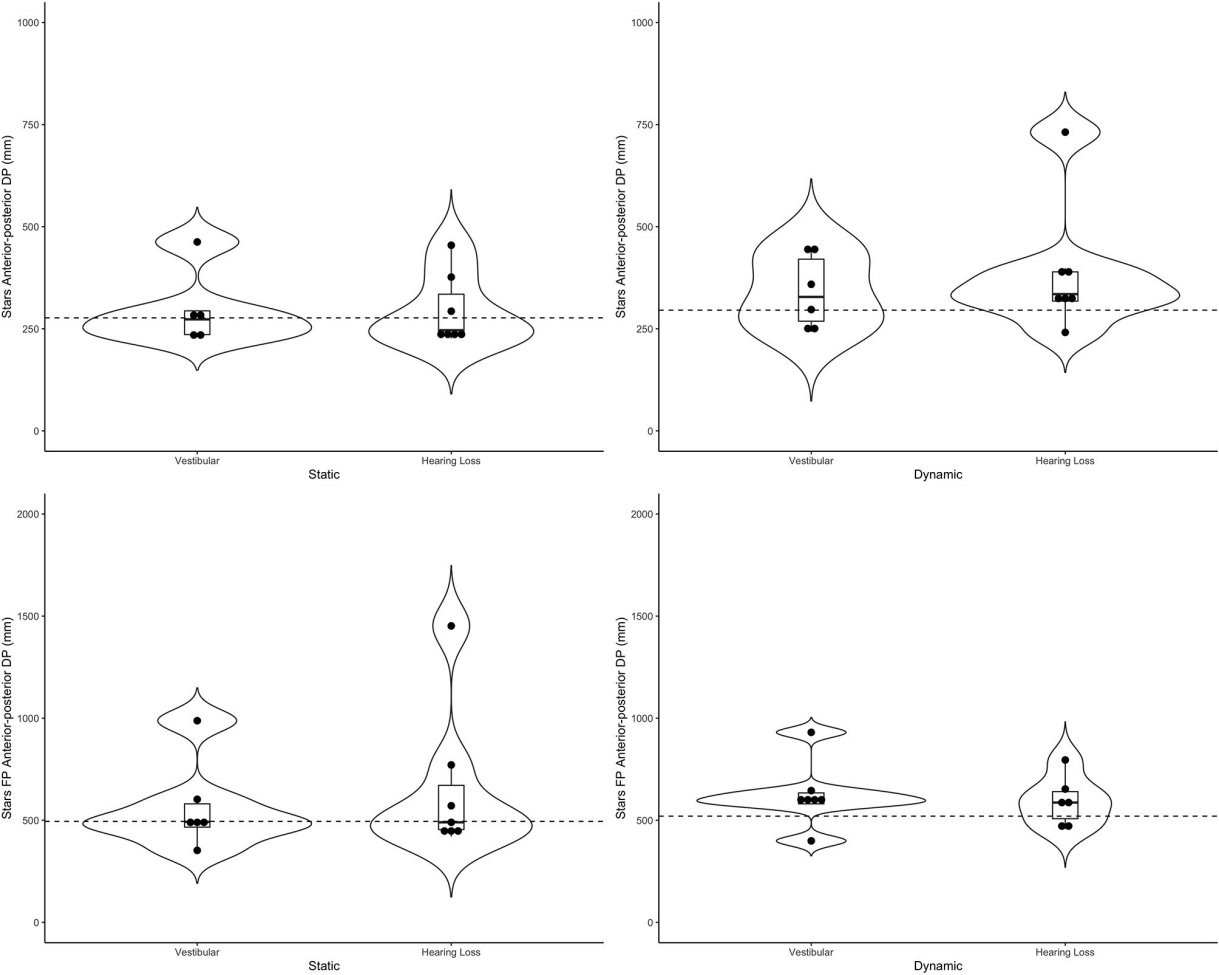
Violin plots representing the distribution of both clinical groups around the mean of the control group (represented by the dashed line) for the stars scene in the anterior-posterior direction. Top plots show head data and bottom plots represent postural sway data (FP = forceplate). Left-hand side represents the static scenes and right-hand side represents the dynamic scenes.

**Figure 6:**
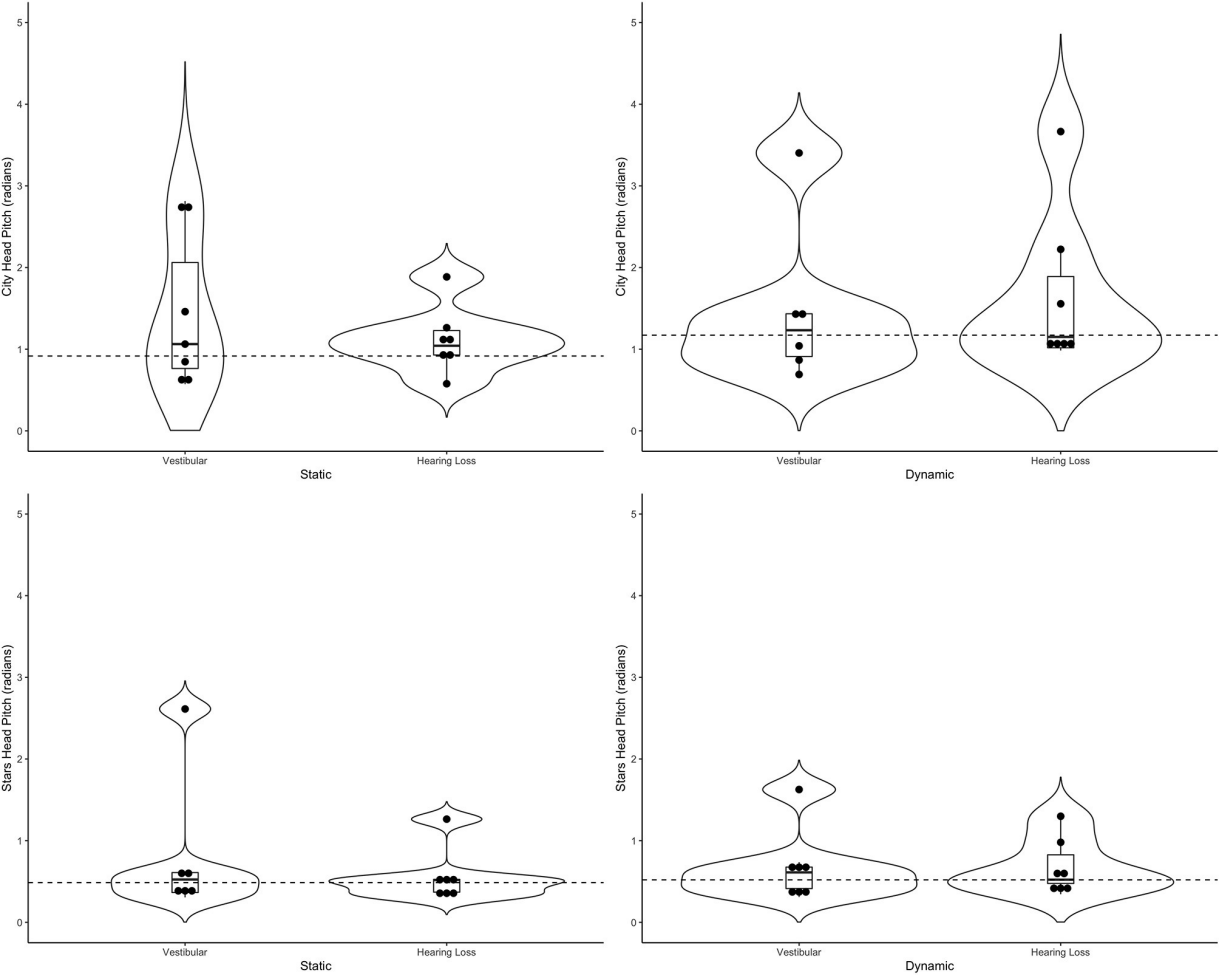
Violin plots representing the distribution head pitch (radians) of both clinical groups around the mean of the control group (represented by the dashed line) for the city scene (top) and stars scene (bottom) Left-hand side represents the static scenes and right-hand side represents the dynamic scenes.

Table 4 includes a detailed description of the clinical groups. Representative violin plots can be seen in Figure 4 (city ML, postural sway, and head), Figure 5 (stars AP, postural sway, and head) and Figure 6 (pitch). All descriptive statistics per group can be found on Appendices A, B.

**Table 4:** Description of the clinical groups.

**Mean (SD) hearing loss onset in years**	**Type of hearing loss**	**Positive bedside vestibular testing**	**History of vestibular rehab**	**History of vertigo**		
**MONAURAL HEARING**						
15.75 (8.61)	4 Bilateral SNHL with a unilateral cochlear implant	1 (2012)	1 (2012)	2 (2012, 2015)		
3 unknown	2 SSD (unamplified)					
	1 SSD + ARHL (unamplified)					
**Mean (SD) vestibular hypofunction onset in years**	**Hearing loss**	**VNG**	**Bedside head thrust**	**Head shaking nystagmus**	**Spontaneous nystagmus without fixation**	**Gaze evoked nystagmus without fixation**
**VESTIBULAR HYPOFUNCTION**						
2.38 (2.59)	1 symmetric bilateral ARHL (unamplified)	4 positive	3 positive	5 positive	2 positive	3 positive
		0 negative	1 negative	2 negative	5 negative	4 negative
		3 NT	3 NT			

*SNHL, Sensorineural Hearing Loss; SSD, Single-sided Deafness (>70 dB, 3-frequency pure-tone average with normal, <25 dB PTA contralateral ear); ARHL, Age-related Hearing Loss (SNHL beginning at 4 KHz with normal, <25 dB PTA in lower frequencies); NT, Not Tested*.

Generally speaking, the majority of the vestibular group moved more than controls when the scenes were dynamic, particularly in the city scene. The monaural hearing group was more diverse. While 4 out of 7 performed similarly to controls, 3 patients emerged outside of the group on dynamic scenes. Of the 3, 1 had prior vestibular rehab, 1 had reduced hearing compared to the rest of the sample. Two of the 3 were elderly CI users (above 70 years of age), but 2 other younger CI users performed similarly to controls. Two of the three had the worst DHI (above 30 when the rest of the group was at 0) and ABC (the only 2 below 90%) scores (for averages see Table 2).

## Discussion

This pilot study provided insights into the inquiry of sounds for postural control, the importance of context, and provided further support for head kinematics as an important additional metric (32, 33) that goes beyond the information provided by postural sway alone. The headset used for this scientific inquiry, the Oculus Rift, allows for simultaneous manipulation of high-quality visuals and sounds while obtaining accurate head kinematics. The study also generated hypotheses for future research investigating postural control in people with monaural hearing and contextual sensory integration in people with vestibular loss.

The first question of this study was how postural sway and head kinematics change in healthy adults in response to auditory perturbations when combined with visual perturbations. We expected to see changes in postural sway between the “low” and “high” visual environments based on previous research using similar visual conditions (38, 40). We also hypothesized that head movement will increase with the sound perturbations. However, these hypotheses were not met as in the current study, the “low” and “high” levels of auditory and visual stimulation did not make a difference in participants' movement when they were standing on the floor with their feet hip-width apart (based on descriptive statistics as well as median-based comparisons not shown). By combining the “low” and “high” data, we were able to explore the role of context, but no longer study the contribution of sounds to balance alone. It is possible that the addition of sounds, which were developed for this study, masked those differences. While all patients noticed the subtle differences between the dynamic scenes, most healthy adults could only detect a difference between the static and dynamic environments.

Our second question was whether the response to sensory perturbations (*via* postural sway and head kinematics) varies by context. Several observations with respect to this question should be discussed. First, medio-lateral postural sway increased with sensory perturbations in the city scene more than the stars scene. Anterior-posterior postural sway increased with sensory perturbations similarly in both environments. This is probably because the visual perturbation in both environments was in the anterior-posterior plane (flow of people or stars), and so a greater response in this direction is expected with an increased visual weight (51). As expected, further insights into people's motor behavior can be obtained from the head segment. For both medio-lateral and anterior-posterior head directional path we observed less movement on the static city environment vs. the static stars. This could be explained by the fact that a static street may feel more natural to people as compared with the “space” feeling many participants reported within the stars scene. This finding highlights the importance of including a static baseline scene to every environment to be studied. Interestingly, the transition from static to dynamic perturbations was larger in the city scene. Our sample also showed significantly more head pitch and roll on the city vs. stars environment. Potentially the participants were more influenced by the moving avatars than the moving stars, and perhaps felt a need to look away or avoid the flow of avatars that were moving toward them.

Our clinical sample was small and diverse, and the analysis of this sample was exploratory. Vestibular and auditory anatomy are closely linked (52), and peripheral vestibular hypofunction is often accompanied by various degrees of hearing loss. The current study did not include diagnostic vestibular testing on patients with hearing loss, so it is possible that these patients with hearing loss had an undiagnosed (or well-compensated) vestibulopathy. Patients with vestibular hypofunction were recruited from a physical therapy clinic whereas patients with monaural hearing loss were recruited from the physician's office where they were seen for their hearing. Patients with vestibular hypofunction also had higher level of simulator sickness than the other 2 groups, particularly after testing. Clinically, diagnostic vestibular testing is not routinely done in people with hearing loss unless they complain of dizziness. Interestingly, the two patients with monaural hearing loss that consistently showed much larger movement in response to sensory perturbation than the rest of the group (particularly AP postural sway and head movement, mostly in the stars scene) were the oldest in the group and had the worst DHI and ABC scores. It is possible that issues related to balance and hearing loss emerge in older age. It also suggests that all people with hearing loss should be regularly queried regarding dizziness and balance. The vestibular group had increased ML postural sway and head movement, particularly in the dynamic city scene. Even though the visual flow of the avatars was in the AP direction, it is possible that patients with vestibular dysfunction attempted to avoid collision by increasing lateral movement, even more than controls did, despite the fact that they were not asked to do so. All participants were asked to “do whatever feels naturally to them to maintain their balance.” Anecdotally, patients in the vestibular group were more likely to report some fear and discomfort with the avatars walking toward them (see Supplementary Video). In our prior work, we observed that participants with unilateral vestibular hypofunction had larger head movement than controls on different dynamic scenes (32, 33) but we did not include a static scene. The current pilot work suggests that larger head movement in the vestibular group particularly occurred in response to the dynamic scene and is not a constant difference (movement was closer to controls on the static scenes). It is important to consider that the descriptive differences observed between the clinical groups and controls were seen despite the fact that the current protocol was done when the patients were standing on a stable surface in a comfortable hips-width stance and the visual perturbations themselves were quite mild. Therefore, any behavior observed in response to the dynamic scenes could be potentially interpreted as excessive visual dependence associated with other sensory loss. To tap into somatosensory dependence we could add a challenging support surface to the paradigm (53). It is likely that further differences would arise between the vestibular group and controls when the surface does not provide stable, reliable somatosensory cues (19).

In addition to the small sample, other design limitations should be mentioned. Given that the study was designed with multilevel sensory load, the static/no sound scene was only repeated once. In the future, we will separate the auditory load from the visual load and include abstract as well as ecological sounds and perform the same number of repetitions on all scenes. Because previous studies did not find loudness/volume of the sound to be a factor in postural sway (6, 54–57), we used the same volume with a range of 62 db (stars) to 68 db (city) for all participants. It is therefore possible that some participants, particularly in the hearing loss group, were not impacted by the addition of the sounds because the sounds were not loud enough for them. In the future, in order to assess individual responses to sounds we will project the sounds at the “loudest that is still comfortable” level. Baseline levels of postural sway and head movement without an HMD were not obtained and could further contribute to the interpretation of the data. The lack of diagnostic vestibular testing on all groups is a limitation as well and needs to be added in future studies.

## Conclusion And Future Research

The current settings were too subtle to test differences between responses to visuals and sounds. Future studies should isolate each modality at the presence of the other. Our data show the importance of context in the study of sensory integration and the feasibility of an HMD setup to do so. In addition, a static baseline scene should be included for each environment. We hypothesize that a varying context will show particular importance in people with vestibular disorders who may be anxious due to the flow of avatars in the immersive contextual environment. The head is an extension of postural sway and responses to HMD-derived sensory perturbations will be magnified around the head segment. While the clinical importance of this observation should be further investigated, it currently appears that HMD-based head kinematics augment data derived from a force platform, and could potentially provide a distinct characteristic of people with vestibular loss. This should be further studied, potentially in combination with electromyography (EMG) of the neck muscles. Fall risk in people with hearing loss has been shown in older adults and our pilot data suggest balance impairments in people with single-sided hearing are more likely to arise in older participants with moderate DHI scores. Future studies utilizing HMDs should further assess aging with and without hearing loss and its impact on postural performance in a larger sample with a more cohesive diagnosis. Vestibular testing needs to be conducted for a clear separation between vestibular-related and hearing-related pathologies and to clarify whether observed performance deficits relate to an underlying vestibular problem or directly to their hearing loss.

## Data Availability Statement

The datasets generated for this study are available at: Lubetzky, Anat (2020), “Auditory Pilot Summer 2020”, Mendeley Data, v1 http://dx.doi.org/10.17632/9jwcp78xzf.1.

## Ethics Statement

The studies involving human participants were reviewed and approved by Institutional Review Board of Mount Sinai; The New York University Committee on Activities Involving Human Subjects. The patients/participants provided their written informed consent to participate in this study.

## Author Contributions

AL, JK, BH, DH, and MC: substantial contributions to the conception or design of the work, or interpretation of data for the work. JK, BH, and MC: recruitment of participants. AL: data acquisition and drafting the work. DH and JL: statistical analysis of the data. All authors revising the work critically for important intellectual content, final approval of the submitted version, and agreement to be accountable for all aspects of the work.

## Conflict of Interest

MC reported unpaid participation in research on cochlear implants and other implantable devices manufactured by Advanced Bionics, Cochlear Americas, MED-El, and Oticon Medical outside the submitted work. The remaining authors declare that the research was conducted in the absence of any commercial or financial relationships that could be construed as a potential conflict of interest.
