# Supplementary material for: Postural and Head Control Given Different Environmental Contexts
Source: Front Neurol. 2021 Jun 3;12:597404. doi: 10.3389/fneur.2021.597404 (PMC8209382; doi:10.3389/fneur.2021.597404)
Supplement: Supplementary file 1 [file Data_Sheet_1.docx]

**Appendix A.** Descriptive Statistics for the ‘Stars’ Scene

|  |  | Static | | | Dynamic | | |
| --- | --- | --- | --- | --- | --- | --- | --- |
|  |  | Control | Monaural Hearing | Vestibular Hypofunction | Control | Monaural Hearing | Vestibular Hypofunction |
| Postural Sway ML (mm) | Mean  (SD) | 312.05  (91.88) | 312.20  (107.9) | 326.23  (127.1) | 312.33  (81.17) | 359.64  (158.7) | 376.15  (150.8) |
|  | Median | 270.55 | 275.43 | 271.65 | 292.19 | 301.50 | 349.47 |
|  | Range | 399.26 | 287.00 | 337.48 | 454.04 | 808.87 | 997.29 |
| Postural Sway AP  (mm) | Mean  (SD) | 494.35  (86.74) | 658.88  (369.5) | 565.25  (222.2) | 519.21  (90.43) | 793.03  (613.6) | 623.96  (201.9) |
|  | Median | 468.76 | 489.74 | 492.06 | 495.58 | 583.41 | 591.27 |
|  | Range | 292.43 | 1031.95 | 635.25 | 430.15 | 2933.66 | 854.16 |
| Head ML  (mm) | Mean  (SD) | 170.42  (56.19) | 176.09  (68.7) | 244.43  (173.3) | 168.90  (51.81) | 184.71  (56.8) | 258.18  (131.8) |
|  | Median | 171.43 | 150.12 | 176.82 | 162.15 | 171.52 | 229.40 |
|  | Range | 210.13 | 174.73 | 453.32 | 335.26 | 317.67 | 663.98 |
| Head AP  (mm) | Mean  (SD) | 276.67  (69.89) | 295.37  (87.9) | 453.17  (385.1) | 295.41  (65.70) | 372.98  (169.8) | 470.50  (310.0) |
|  | Median | 275.92 | 246.34 | 283.46 | 290.90 | 335.20 | 354.41 |
|  | Range | 269.79 | 174.73 | 453.32 | 402.19 | 317.67 | 663.98 |
| Head Pitch  (radians) | Mean  (SD) | 0.48  (0.15) | 0.55  (0.33) | 0.82  (0.89) | 0.52  (0.17) | 0.64  (0.38) | 0.75  (0.50) |
|  | Median | 0.47 | 0.52 | 0.52 | 0.48 | 0.52 | 0.60 |
|  | Range | 0.60 | 0.95 | 2.31 | 1.30 | 1.86 | 2.47 |
| Head Yaw  (radians) | Mean  (SD) | 0.39  (0.12) | 0.42  (0.21) | 0.55  (0.45) | 0.42  (0.14) | 0.48  (0.24) | 0.51  (0.30) |
|  | Median | 0.37 | 0.36 | 0.30 | 0.39 | 0.42 | 0.42 |
|  | Range | 0.53 | 0.62 | 1.10 | 1.00 | 1.49 | 1.88 |
| Head Roll  (radians) | Mean  (SD) | 0.31  (0.11) | 0.33  (0.20) | 0.52  (0.55) | 0.33  (0.11) | 0.34  (0.13) | 0.41  (0.23) |
|  | Median | 0.30 | 0.24 | 0.29 | 0.31 | 0.32 | 0.35 |
|  | Range | 0.42 | 0.58 | 1.41 | 0.62 | 0.75 | 1.05 |
