# Supplementary material for: Postural and Head Control Given Different Environmental Contexts
Source: Front Neurol. 2021 Jun 3;12:597404. doi: 10.3389/fneur.2021.597404 (PMC8209382; doi:10.3389/fneur.2021.597404)
Supplement: Supplementary file 2 [file Data_Sheet_2.docx]

**Appendix B.** Descriptive Statistics for the ‘City’ Scene

|  |  | Static | | | Dynamic | | |
| --- | --- | --- | --- | --- | --- | --- | --- |
|  |  | Control | Monaural Hearing | Vestibular Hypofunction | Control | Monaural Hearing | Vestibular Hypofunction |
| Postural Sway ML  (mm) | Mean  (SD) | 284.07  (53.06) | 345.52  (98.7) | 371.90  (139.8) | 331.27  (92.52) | 400.88  (211.4) | 514.75  (385.6) |
|  | Median | 269.67 | 306.02 | 375.89 | 309.42 | 311.30 | 370.57 |
|  | Range | 213.33 | 226.26 | 407.97 | 682.05 | 991.59 | 1703.08 |
| Postural Sway AP  (mm) | Mean  (SD) | 488.88  (62.70) | 657.27  (263.6) | 648.62  (244.1) | 539.40  (106.56) | 748.63  (386.3) | 708.38  (261.1) |
|  | Median | 472.47 | 557.62 | 631.50 | 515.77 | 642.97 | 643.40 |
|  | Range | 220.71 | 728.98 | 740.06 | 743.42 | 2464.33 | 1177.60 |
| Head ML  (mm) | Mean  (SD) | 135.69  (26.39) | 166.27  (53.9) | 211.16  (91.0) | 186.56  (67.26) | 250.29  (174.6) | 344.65  (379.7) |
|  | Median | 134.32 | 163.14 | 208.16 | 180.57 | 187.31 | 228.56 |
|  | Range | 102.37 | 131.55 | 252.53 | 450.88 | 876.77 | 1869.49 |
| Head AP  (mm) | Mean  (SD) | 214.03  (40.31) | 246.45  (80.7) | 387.29  (285.7) | 277.48  (72.54) | 359.30  (154.4) | 446.03  (294.4) |
|  | Median | 215.27 | 236.25 | 256.76 | 281.25 | 323.90 | 336.88 |
|  | Range | 165.11 | 131.55 | 252.53 | 402.50 | 876.77 | 1869.49 |
| Head Pitch  (Rad) | Mean  (SD) | 0.92  (0.36) | 1.12  (0.41) | 1.44  (0.93) | 1.17  (0.39) | 1.57  (1.00) | 1.93  (1.79) |
|  | Median | 0.86 | 1.04 | 1.06 | 1.10 | 1.20 | 1.27 |
|  | Range | 1.79 | 1.31 | 2.24 | 2.91 | 4.29 | 9.55 |
| Head Yaw  (Rad) | Mean  (SD) | 0.79  (0.28) | 0.99  (0.50) | 1.76  (1.94) | 0.97  (0.40) | 1.46  (1.34) | 2.18  (3.44) |
|  | Median | 0.72 | 0.89 | 0.96 | 0.88 | 1.01 | 1.07 |
|  | Range | 1.30 | 1.51 | 5.48 | 3.03 | 6.09 | 20.01 |
| Head Roll  (Rad) | Mean  (SD) | 0.57  (0.16) | 0.74  (0.36) | 0.88  (0.41) | 0.74  (0.28) | 0.91  (0.67) | 1.35  (1.70) |
|  | Median | 0.53 | 0.61 | 0.68 | 0.70 | 0.70 | 0.92 |
|  | Range | 0.57 | 1.05 | 1.01 | 2.18 | 3.36 | 10.04 |
